# Eleven-Year Report of High Number of Diphtheria Cases in Children in East Java Province, Indonesia

**DOI:** 10.3390/tropicalmed9090204

**Published:** 2024-09-07

**Authors:** Dominicus Husada, Yustika Hartini, Kalista Wahyu Nuringhati, Sandy Grace Tindage, Rahma Ira Mustikasari, Leny Kartina, Dwiyanti Puspitasari, Parwati S. Basuki, Ismoedijanto Moedjito, Zumaroh Zumaroh, Hugeng Susanto, Wahyu Wulandari, Sulvy Dwi Anggraini, Erwin Astha Triyono

**Affiliations:** 1 Department of Child Health, Faculty of Medicine, Universitas Airlangga, Surabaya 60132, Indonesia; rahmaira.unair@yahoo.com (R.I.M.); lenykartina@yahoo.com (L.K.); dwiyanti-p@fk.unair.ac.id (D.P.); parwatisetiono@yahoo.com (P.S.B.); ismoemp@gmail.com (I.M.); 2 Department of Child Health, Dr. Soetomo General Academic Hospital, Surabaya 60286, Indonesia; yustikahrtn@gmail.com (Y.H.); kalista.wahyu@gamil.com (K.W.N.); sandygtindage@gmail.com (S.G.T.); 3 East Java Provincial Health Office, Surabaya 60231, Indonesia; zuma.survim.dinkesjatim@gmail.com (Z.Z.); hugengsusanto492@gmail.com (H.S.); wawulandari@gmail.com (W.W.); sulvy_da@yahoo.com (S.D.A.); erwintriyono@yahoo.com (E.A.T.); 4 Department of Internal Medicine, Faculty of Medicine, Universitas Airlangga, Surabaya 60115, Indonesia; 5 Department of Internal Medicine, Dr. Soetomo General Academic Hospital, Surabaya 60286, Indonesia

**Keywords:** diphtheria, children, adolescent, East Java province, Indonesia, surveillance, vaccine-preventable disease, communicable disease

## Abstract

A high incidence of diphtheria cases in children in East Java province, Indonesia, has been observed since the beginning of this century. Despite many efforts, the outbreaks continue. This study aims to explain the high incidence of diphtheria in children in East Java province since 2013. This cross-sectional surveillance report-based study used data from 38 districts in East Java since 1 January 2013. Collected data included demographics, clinical information, additional examinations, immunization history, and close contact management. Over eleven years, there were 4009 diphtheria patients, of whom 2921 (72.86%) were under 18 years of age. Boys (59.77%) outnumbered girls, and the most common age category was >60–144 months (51.66%). Most cases had incomplete or zero immunization (76.16%). Tonsillopharyngeal diphtheria was the most common type (69.60%). The five top districts with the most cases were Surabaya, Sidoarjo, Kabupaten Blitar, Kota Malang, and Kabupaten Malang. The eleven-year case fatality rate (CFR) was 2.36% (69/2921). This study shows that diphtheria cases in children and adolescents in East Java have consistently been high, and low immunization coverage might still be the leading cause. There has also been a shift in the district distribution. Diphtheria outbreaks require complete and sustainable efforts, not just outbreak response immunizations.

## 1. Introduction

High numbers of diphtheria cases have been recorded in Indonesia since 2011 [1,2,3]. Despite the national immunization program initiated in the 1980s, coverage in some areas remains low, contributing to the persistence of several infectious diseases, including diphtheria. At the end of the previous century, the number of diphtheria cases decreased, but since 2005, the number has increased [1,2,3]. Currently, a high number of diphtheria cases worldwide have been recorded in the World Health Organization (WHO) Southeast Asia Regional Office (SEARO) and Africa Regional Office (AFRO) regions. Indonesia usually ranks second after India in the number of children with diphtheria [4,5].

Diphtheria is a dangerous contagious disease caused by *Corynebacterium diphtheriae*, *C. ulcerans*, and *C. pseudotuberculosis*. In the developed world, most cases nowadays are caused by *C. ulcerans*, but in developing countries, *C. diphtheriae* still plays a significant role. All clinical signs and symptoms of diphtheria are caused by an exotoxin, with a pseudomembrane as the hallmark. The main prevention is immunization using diphtheria toxoid. The vaccine targets the toxin rather than the bacteria [6,7,8,9,10].

Among all pseudomembrane locations, the tonsils and pharynx are the most common. Children with diphtheria usually have low-grade fever, anorexia, malaise, and sore throat. Nasal and skin diphtheria can show location-based signs and symptoms as well. The pseudomembrane is dirty, brownish-white in color, unified, and difficult to detach without bleeding. The most crucial additional examination is microbiological culture from the location of the pseudomembrane or a polymerase chain reaction (PCR) test. Treatment requires antibiotics, diphtheria antitoxin, immunization, and contact tracing. Without proper and prompt treatment, diphtheria can be fatal. The most common complications are myocarditis, nephritis, and neuritis, besides local airway obstruction [6,7,8,9,11].

Among the 38 provinces in Indonesia, East and West Java are the most prominent, contributing more than half of all cases each year [2,12,13]. The lowest vaccine coverage was recorded in Aceh and West Sumatra provinces, but due to their limited population, the role of the two provinces in Java Island is more significant. The population in East Java, the second most populated province in Indonesia, is 35 million, with approximately 15% of children and adolescents under 18 years of age. This province has 38 districts and serves as the tertiary referral center for health and medical services in eastern Indonesia.

Surveillance of vaccine-preventable diseases such as diphtheria is crucial as part of the government’s and society’s efforts to address the problem. In Indonesia, surveillance for vaccine-preventable diseases has declined continuously, especially after the political turmoil in 1998, due to several reasons. Surveillance reports were usually made at the village level and collected routinely, with varying completeness and accuracy [1]. This study aims to present data from 13 years of high diphtheria cases in East Java province.

## 2. Materials and Methods

### 2.1. Study Design, Population, and Data Collection

This cross-sectional surveillance report is based on routine data collection from villages and districts. All 38 District Health Offices in East Java province provide routine reports on a weekly and monthly basis. Health officers can also send daily reports under exceptional circumstances. Several items in the reports include name, sex, date of birth or age, address, date of diagnosis, date of initial signs and symptoms, all signs and symptoms, physical examination results, laboratory reports, radiology examinations if available, birth history, history of immunization, history of medication, nutritional and growth development history, location of treatment, medication provided, length of stay in clinics or hospitals, microbiological culture results, data on close contacts, and follow-up after hospitalization. Once the health officer receives information about diphtheria patients, the local team traces the patients, visits their homes and neighborhoods, monitors surrounding areas, and traces close contacts. By definition, close contacts include all family members in the same house, classmates in the same room, and close friends. All final data are collected at the East Java Provincial Health Office. Data from this provincial office are later sent to the ministry in Jakarta.

The inclusion criteria for this report were age less than 18, a diagnosis of diphtheria, residence in East Java province, and data recorded at the East Java Provincial Health Office. Children or adolescents with incomplete data were excluded. All data from 2013 to 2023 were used and analyzed.

### 2.2. Diagnosis Criteria

The Indonesian Ministry of Health set modified criteria for diagnosing diphtheria, in which a child can be diagnosed with diphtheria if the microbiological culture shows toxigenic *Corynebacterium diphtheriae* or if the National Diphtheria Expert Committee (NDEC) decides on a diphtheria diagnosis, regardless of the microbiological culture result [14]. The clinical signs and symptoms should support the appearance of diphtheria, i.e., low-grade fever, sore throat, anorexia, malaise, and localized signs and symptoms. To make the final decision, the expert committee requires a complete medical record and a picture of the pseudomembrane. According to the regulation, local medical doctors are advised not to make the final diagnosis themselves. In this report, cases without positive microbiological culture or approval from the NDEC were excluded.

### 2.3. Laboratory

Until 2024, only three laboratories in Indonesia were capable of performing complete microbiological cultures. One of them, the Public Health Laboratory (BBLKM), is in Surabaya, the capital of East Java province. All other laboratories in Indonesia could not perform the toxigenicity test. The microbiological culture was performed according to the WHO Laboratory Guidelines [15,16]. Swab specimens from the nose and throat were placed in Amies media and then sent to the laboratory the same day. The final result of a positive culture had to mention the toxigenicity test by Modified Elek and the biovar. Until 2024, the laboratories in Indonesia only looked for *C. diphtheriae*.

### 2.4. Case Management

According to national guidelines, every positive diphtheria case requires hospitalization for at least ten days. Isolation procedures, proper antibiotics, diphtheria antitoxin, and contact tracing should be performed. In Indonesia, penicillin procaine and erythromycin are the two available antibiotics. Azithromycin, also recommended by the WHO, is not widely used. Currently, worldwide, there is a shortage of diphtheria antitoxin [17,18]. The Ministry of Health decided to distribute this drug centrally. All DAT will be distributed only from the capital city for cases with approval from the NDEC.

### 2.5. Data Analysis

In this report, the statistical analyses used SPSS version 19. Categorical variables are reported as numbers and percentages of patients. Case fatality rates (CFRs) were calculated. A pie chart is used to display the comparison of cases before and during the COVID-19 pandemic. The map of East Java province also shows the top five districts in terms of the highest number of cases.

### 2.6. Ethics

Dr. Soetomo General Hospital’s Health Research Ethical Committee approved the exemption letter for this study. Informed consent was also waived as the study was based on routine surveillance reports and would not disclose any individual data.

## 3. Results

### 3.1. Total Cases, Districts, and Mortality

During the 11 years, there were 4009 diphtheria patients, and 2921 (72.86%) were less than 18 years of age. The CFR for the total cases was 2.4% (96/4009). Table 1 describes the diphtheria cases by districts and outcome, focusing on patients less than 18 years old. In general, children were more prevalent than adults; the most prevalent age group was 5 to 12. The eleven-year CFR of the less-than-18-year-old group was 2.36% (69/2921).

**Figure 1:**
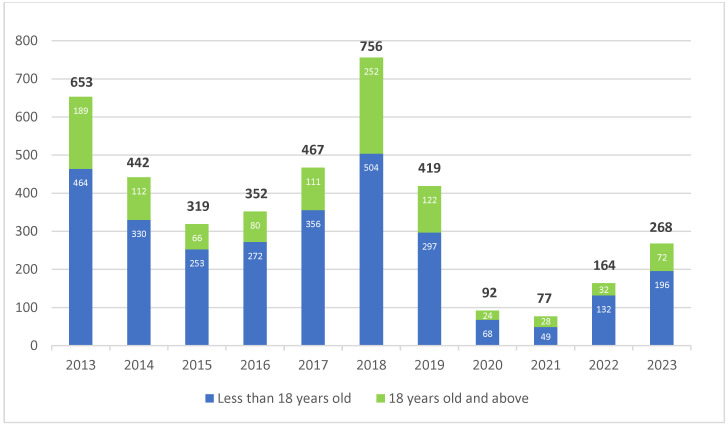
The distribution of diphtheria cases in East Java by district and age group 2013–2023. x-axis: year; y-axis: number of patients; number in black: total diphtheria patients in that particular year.

**Figure 2:**
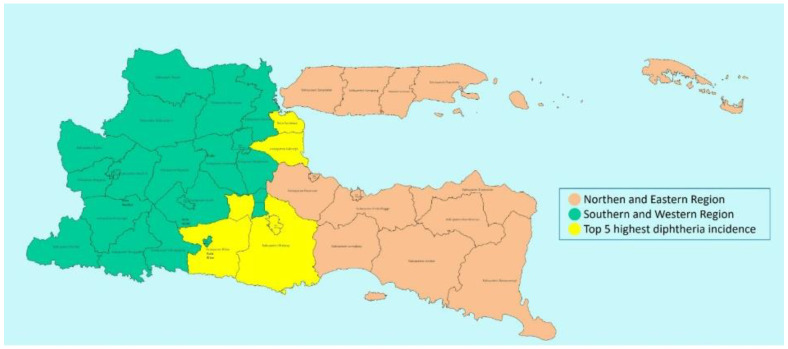
Comparison of cases between the northern and eastern vs. southern and western parts of the East Java province. North and east = 1159 cases vs. south and west = 1762 cases (=2:3).

Figure 1 describes the total cases for both age groups: those under 18 and those 18 and above. Figure 2 shows the comparison of cases between the northern and eastern vs. southern and western parts of the province. Child and adolescent diphtheria cases were found in all 38 districts in East Java province. The top five districts regarding the total number of cases were Surabaya, Sidoarjo, Kabupaten Blitar, Kota Malang, and Kabupaten Malang.

### 3.2. Cases by Age, Sex, Immunization Status, and Pseudomembrane Location

Table 2 shows the cases by sex, age categories, immunization status, and the location of the pseudomembrane. On the basis of the immunization status, most cases did not receive complete immunization doses. Around 71.48% (2088/2921) of cases could not show valid proof of immunization records, such as immunization cards or official records from community health centers, doctors, or midwives, and solely used the parents’ recall.

Tonsil and/or pharyngeal diphtheria was the most prevalent compared to other locations. These patients reported that sore throat (79.05% or 2309/2921) and low-grade fever (94.11% or 2749/2921) were the most predominant signs and symptoms. Only 12.29% (359/2921) of cases had positive microbiological results of toxigenic *Corynebacterium diphtheriae*, dominated by the biotype mitis. Almost all cases were hospitalized (97.91% or 2860/2921).

### 3.3. Number of Diphtheria Cases before and during the Pandemic

**Figure 3:**
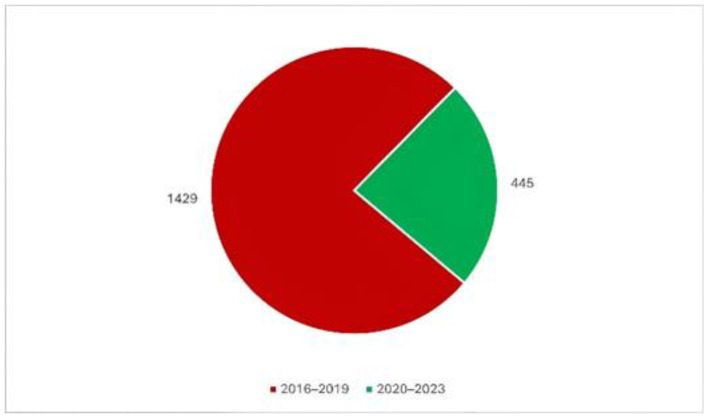
Comparison of diphtheria cases in children before and during the pandemic (4-year period).

Figure 3 shows the comparison between the number of diphtheria cases before and during the COVID-19 pandemic (four years for each period). The COVID-19 pandemic started in 2020, and the number of cases significantly declined, but in 2022–2023, there was a resurgence. As shown in this figure, the total number of cases during 2016–2019 was 3.2 times more than the cases in 2020–2023 (or 1429/445).

## 4. Discussion

Data from the WHO website showed the total number of diphtheria cases in Indonesia (all ages) for ten years, from 2013 until 2022 (data from the year 2015 were missing from the website), with as many as 5056 patients [4]. As shown in this report, cases from East Java province were predominant compared to the overall number of patients in Indonesia.

The high number of diphtheria cases in East Java province has reemerged since 2005 in Madura Island, one of the areas with many outbreaks in recent years. The peak was in 2009. Most cases were below 18 years old, especially 15 years or less. Traditionally and previously, most patients came from the northern and eastern parts of the province. In this area, the vaccination coverage was chronically low in several pockets until today [13,19]. Based on the official government data, the overall coverage, especially for primary immunization, was actually good enough, but the booster always needs improvement. Outbreak response immunization can cover this booster problem [20].

In most outbreaks of vaccine-preventable diseases, the leading cause was low vaccination coverage [21,22,23,24,25]. Our report shows a similar problem: most children and adolescents with diphtheria had a poor immunization history during this period, which is the primary prevention effort. According to the Indonesian Ministry of Health, children must receive the diphtheria vaccine seven times before they finish elementary school to achieve long-term protection. The unimmune and susceptible person is always the main target of this disease, as shown in many diphtheria outbreaks. The diphtheria toxoid vaccine is more than 100 years old with an excellent safety record [10]. Since the initiation of the vaccination program, the world has significantly reduced these cases in many countries. Although no controlled clinical trial of the efficacy of the diphtheria toxoid in preventing diphtheria has ever been conducted, there were some data from previous observational studies. The effectiveness of diphtheria toxoid, especially in children, can be as high as 95%, as shown in several outbreaks, including in the Newly Independent States or the Soviet Union in the 1990s [10]. Truelove et al. found that the vaccine efficacy of triple complete immunization against symptomatic disease during the first year of life was 87%. It was only 71% if the child only received one or two doses [9]. This vaccine’s effectiveness was lower in adults [10]. The cases with complete immunization by age in this report were somewhat high. This probably happened because of the lack of formal proof of vaccination. Another reason might be the vaccine handling, especially in remote areas; however, so far, there has been no study to prove this possibility.

Outbreaks also have several other risk factors, including cultural differences between the most and least affected areas [19,25]. In this report, districts from southern and western provinces had more cases, and the top five districts and cities regarding the number of cases lie somewhere on the border between the western and eastern parts of the provinces. The higher number of cases in the southern and western areas might be due to at least four factors. First, big districts and cities (such as Surabaya, Sidoarjo, and Malang) had more cases because they had more medical officers with more complete facilities, so they could better identify children with diphtheria. These areas also have medical schools. Second, people in more remote areas seldom visit medical facilities. The distance between their homes and the community health centers or hospitals, the transportation facilities, and the economic condition may be obstacles. Underdiagnosed cases may be the critical reason. Third, the activities of surveillance officers in every district may also play a role. The fourth is the decline of immunization coverage, especially during the pandemic era [26]. In 2022, the Indonesian Ministry of Health also added five more antigens to the national immunization schedule. These additional vaccines insisted that babies receive multiple injections at least five times during the first year of life. Multiple injections create additional difficulty since some refuse them, so the immunization coverage target will be harder to reach.

During the last ten years, several outbreaks of diphtheria have also happened in many countries such as India, Venezuela, Yemen, West Africa, and Nigeria [27,28,29,30,31,32,33,34,35]. In Southeast Asia, these outbreaks have also been found in Laos, Thailand, Malaysia, Myanmar, and even Singapore [22,23,36,37,38,39]. Most of those outbreaks last for a short time. In India, the total number of diphtheria cases for the last several years has also declined.

East Java province’s government has performed several outbreak response immunizations [20]. However, that effort was usually not followed by consistent and continuous strategies to maintain high immunization coverage or to close the immunization gap. Consequently, the number of diphtheria cases has been steadily high. 

In this report, most of the patients were boys. They are considered more active, so the risk of infection is also higher [19]. The tonsils and pharynx were the most common sites of diphtheria worldwide, as found in this report [6,7,8]. The disease is transmitted via droplets [6,7,8]; however, skin diphtheria is responsible for the continuous occurrence of cases [25,40]. Medical personnel often consider ordinary skin treatments such as steroids and antibiotics rather than specific diphtheria management. In East Java, skin diphtheria cases were only 0.41%. Detecting this type of disease is difficult, as its morphology is usually similar to other skin infections. Tonsillopharyngeal diphtheria can easily cause systemic complications in the heart, kidneys, or nervous system [6,7,8]. The most predominant cause of mortality is myocarditis [41,42].

During the COVID-19 pandemic, all infectious diseases were significantly reduced, including vaccine-preventable diseases, especially those transmitted via the respiratory tract [43]. All preventive measures during the pandemic also worked against many diseases [44,45]. These data are consistent with other diseases in many developed countries, such as RSV and influenza [46,47,48]. During the first and second pandemic years (2020–2021), cases were very low due to many social behavior limitations. After that, cases gradually increased over the next two years.

The CFR in this region was relatively low. The average fatality rate from various studies in non-outbreak conditions in many countries was 5–10% [21]. Truelove et al. calculated that the CFR could be as high as 63% in their review [9]. During the diphtheria outbreak in Russia, the CFR was around 2.5–3% [21]. In Nigeria, the CFR was much higher [49,50]. Meanwhile, diphtheria among Rohingya refugees showed a CFR of under 1% [36,37]. Most of the high fatalities during the outbreaks were found in initial cases (usually because of late misdiagnosis) and declined afterwards with proper and prompt actions. Vaccination, isolation, and antibiotics play very significant roles [9]. The case fatality rate in children and low-resource settings remains higher [9]. The high number of cases has been noted for more than ten years in East Java province; thus, many medical officers, even in peripheral areas, are already aware of the prompt and proper management of diphtheria cases despite the problems regarding medical facilities. There is always the possibility of overdiagnosis of some subjects, although in Indonesia, diphtheria experts are already involved in making the diagnosis.

The limitations of this report include the cross-sectional record-based design, manual recording system, and various types of reports at many levels. Our record as the primary data source was imperfect, and many essential data were missing. The consistency among data could not be easily checked and rechecked. Currently, several types of diphtheria reports at different levels of offices have different formats, which adds to the difficulties in keeping the report complete and precise. Digitalization projects to improve data quality and reports initiated by the Ministry of Health are ongoing in Indonesia.

This report has several implications. The East Java Health Office needs to organize a better strategy to address the problem shortly. During the COVID-19 pandemic, immunization coverage declined significantly, and many experts believe upcoming outbreaks are imminent [26,51]. This gap should be filled immediately. The government also needs to involve more experts from non-medical communities, such as cultural and social scientists, to contribute to a broader plan to persuade people in certain unimmunized pockets. Regarding the districts with the highest cases, it is time to reconsider the broader spread of diphtheria, so the southern and western regions should also be prioritized.

## 5. Conclusions

This report shows that diphtheria cases in children and adolescents in East Java over the last 11 years have been high, although the CFR has remained quite low. These cases declined during the pandemic years but then rose again. There was a shift regarding the districts with the highest number of cases. Low immunization coverage may still be the most essential factor in this province. Diphtheria outbreaks require more complete and sustainable efforts, not just outbreak response immunizations. Besides the ORI, the coverage of basic and regular immunization, including booster doses, is the most important aspect.

## Figures and Tables

**Table 1:** Diphtheria cases in children less than 18 years old in East Java 2013–2023 by districts and outcome.

	Years											
	2013	2014	2015	2016	2017	2018	2019	2020	2021	2022	2023	Total
Total number of cases	464	330	253	272	356	504	297	68	49	132	196	2921
Cases by Districts:												
Bangkalan	40	6	14	5	5	18	20	3	1	3	6	121
Banyuwangi	8	9	4	1	5	7	2	0	0	4	0	40
Batu—Kota	2	4	8	3	7	6	8	0	0	1	3	42
Blitar—Kota	8	15	5	15	6	7	3	0	1	0	0	60
Blitar—Kabupaten	17	20	42	45	11	26	24	6	3	10	7	211
Bojonegoro	5	1	7	4	1	10	4	0	1	4	3	40
Bondowoso	11	4	3	0	1	2	1	0	0	1	1	24
Gresik	10	5	9	24	17	15	11	4	3	1	7	106
Jember	34	10	6	4	4	12	9	1	0	2	7	89
Jombang	7	12	1	7	13	17	11	1	2	5	7	83
Kediri—Kabupaten	12	6	6	3	7	20	17	2	0	4	3	80
Kediri—Kota	10	2	0	2	5	8	6	0	0	1	4	38
Lamongan	1	5	2	0	4	11	6	3	0	0	3	35
Lumajang	22	7	1	2	10	38	16	3	6	8	6	119
Madiun—Kabupaten	7	3	2	1	0	7	1	0	0	1	2	24
Madiun—Kota	20	2	0	6	0	6	2	1	0	0	2	39
Magetan	6	4	1	0	2	5	3	0	1	3	4	29
Malang—Kabupaten	13	27	9	4	18	23	27	2	2	4	5	134
Malang—Kota	15	18	14	26	17	18	14	4	0	9	7	142
Mojokerto—Kabupaten	5	16	9	6	8	18	2	6	1	3	8	82
Mojokerto—Kota	9	5	7	5	3	8	3	2	0	4	3	49
Nganjuk	3	0	7	7	20	12	10	4	0	2	7	72
Ngawi	2	4	4	3	6	8	3	1	1	3	0	35
Pacitan	4	2	0	2	0	4	3	0	0	1	2	18
Pamekasan	4	4	3	0	2	11	1	1	0	0	1	27
Pasuruan—Kabupaten	5	2	4	9	28	18	12	2	4	8	6	98
Pasuruan—Kota	1	0	1	2	15	10	6	1	0	4	4	44
Ponorogo	5	0	1	0	3	5	2	0	0	0	0	16
Probolinggo—Kabupaten	10	4	2	4	14	9	4	0	0	5	6	58
Probolinggo—Kota	2	0	1	1	2	6	7	0	0	3	3	25
Sampang	7	8	2	6	33	6	2	1	2	7	34	108
Sidoarjo	31	33	20	20	26	31	17	11	4	10	13	216
Situbondo	5	15	4	5	3	16	4	2	2	3	0	59
Sumenep	8	1	6	4	8	6	4	0	4	2	5	48
Surabaya	77	43	27	27	28	44	18	3	8	11	13	299
Trenggalek	3	8	5	6	4	4	4	1	0	1	2	38
Tuban	2	3	3	3	13	19	5	3	1	1	1	54
Tulungagung	33	22	13	10	7	13	5	0	2	3	11	119
Outcome:												
Survived	447	321	245	266	345	504	295	68	49	126	186	2852
Died (n(%))	17 (3.7)	9 (2.7)	8 (3.2)	6 (2.2)	11 (3.1)	0 (0)	2 (0.7)	0 (0)	0 (0)	6 (4.5)	10 (5.1)	69 (2.4)

**Table 2:** Diphtheria cases in children in East Java 2013–2023 by age, sex, and immunization status.

Variables	Cases (*n*(%))
Sex:	
Girls/females	1175 (40.23)
Boys/males	1746 (59.77)
Age Category (months):	
Less than 12 months	44 (1.51)
12–36 months	323 (11.06)
>36–60 months	586 (20.06)
>60–144 months	1509 (51.66)
>144–<196 months	459 (15.71)
Immunization:	
Unimmunized	444 (15.20)
Not completed by age	1780 (60.94)
Completed by age	600 (20.54)
Unknown	97 (3.32)
Location of the diphtheria:	
Tonsil—pharyngeal	2033 (69.60)
Tonsillar	12 (0.41)
Pharyngeal	466 (15.95)
Laryngeal	25 (0.86)
Nasal	4 (0.14)
Skin	12 (0.41)
No data	369 (12.63)

## Data Availability

The data that support the findings of this report are available from the East Java Provincial Health office upon reasonable request.
